# *In situ *aromatase expression in primary tumor is associated with estrogen receptor expression but is not predictive of response to endocrine therapy in advanced breast cancer

**DOI:** 10.1186/1471-2407-9-185

**Published:** 2009-06-16

**Authors:** Anne E Lykkesfeldt, Katrine L Henriksen, Birgitte B Rasmussen, Hironobu Sasano, Dean B Evans, Susanne Møller, Bent Ejlertsen, Henning T Mouridsen

**Affiliations:** 1 Department of Tumor Endocrinology, Institute of Cancer Biology, Danish Cancer Society, DK- 2100 Copenhagen Ø, Denmark; 2 Department of Pathology, Herlev Hospital, DK-2730, Denmark; 3 Department of Pathology, Tohoku University School of Medicine, Sendai 980-8575, Japan; 4 Novartis Institutes for BioMedical Research, CH-4002 Basel, Switzerland; 5 Danish Breast Cancer Cooperative Group, Rigshospitalet, DK- 2100 Copenhagen Ø, Denmark; 6 Department of Oncology, Rigshospitalet, DK- 2100 Copenhagen Ø, Denmark; 7 Danish Breast Cancer Cooperative Group, Rigshospitalet, DK- 2100 Copenhagen Ø, Denmark

## Abstract

**Background:**

New, third-generation aromatase inhibitors (AIs) have proven comparable or superior to the anti-estrogen tamoxifen for treatment of estrogen receptor (ER) and/or progesterone receptor (PR) positive breast cancer. AIs suppress total body and intratumoral estrogen levels. It is unclear whether *in situ *carcinoma cell aromatization is the primary source of estrogen production for tumor growth and whether the aromatase expression is predictive of response to endocrine therapy. Due to methodological difficulties in the determination of the aromatase protein, COX-2, an enzyme involved in the synthesis of aromatase, has been suggested as a surrogate marker for aromatase expression.

**Methods:**

Primary tumor material was retrospectively collected from 88 patients who participated in a randomized clinical trial comparing the AI letrozole to the anti-estrogen tamoxifen for first-line treatment of advanced breast cancer. Semi-quantitative immunohistochemical (IHC) analysis was performed for ER, PR, COX-2 and aromatase using Tissue Microarrays (TMAs). Aromatase was also analyzed using whole sections (WS). Kappa analysis was applied to compare association of protein expression levels. Univariate Wilcoxon analysis and the Cox-analysis were performed to evaluate time to progression (TTP) in relation to marker expression.

**Results:**

Aromatase expression was associated with ER, but not with PR or COX-2 expression in carcinoma cells. Measurements of aromatase in WS were not comparable to results from TMAs. Expression of COX-2 and aromatase did not predict response to endocrine therapy. Aromatase in combination with high PR expression may select letrozole treated patients with a longer TTP.

**Conclusion:**

TMAs are not suitable for IHC analysis of *in situ *aromatase expression and we did not find COX-2 expression in carcinoma cells to be a surrogate marker for aromatase. *In situ *aromatase expression in tumor cells is associated with ER expression and may thus point towards good prognosis. Aromatase expression in cancer cells is not predictive of response to endocrine therapy, indicating that *in situ *estrogen synthesis may not be the major source of intratumoral estrogen. However, aromatase expression in combination with high PR expression may select letrozole treated patients with longer TTP.

**Trial registration:**

Sub-study of trial P025 for advanced breast cancer.

## Background

Treatment with the non-steroidal antiestrogen (AE) tamoxifen has been the first-line endocrine treatment of choice for breast cancer patients for more than 30 years. However, the third-generation aromatase inhibitors (AIs) anastrozole, letrozole and exemestane have in large randomized trials shown to be comparable or superior to tamoxifen as treatment for postmenopausal women with hormone receptor (HR) positive metastatic breast cancer [1-6]. The objective response rates ranged from 21% to 33% with clinical benefit rate varying between 49% and 59% [1,2,4], necessitating improvements in treatments and development of response predictors to the different options.

Expression of the estrogen receptor (ER) is a prerequisite for estrogen dependent tumor growth and ER positivity in the primary tumor has been used as a selection criterion for endocrine therapy since 1975 [7]. Furthermore, ER is also well known to be an important prognostic factor, indicating good prognosis [8]. The progesterone receptor (PR) is an estrogen-inducible protein and improved response rates have been seen in tumors, which besides ER, also express PR [9-11], with increasing ER and PR scores being associated with better response to tamoxifen in ER positive metastatic breast cancer [12]. Today, most laboratories perform immunohistochemical (IHC) determination of both ER and PR and a good correlation between the quantitative level determined with the classical ligand-binding assays and the immunohistochemical scores have been found for both ER and PR [12].

The third-generation AIs anastrozole, letrozole and exemestane suppress total-body aromatization by 98%, more than 99% and 98%, respectively [13,14], demonstrating the superior suppressive potency of these drugs compared to the previous first- and second-generation compounds [15]. Furthermore in studies with direct measurements of estrogen levels in tumor tissue, AI treatment resulted in nearly complete suppression of intratumoral estrogen levels [16-20]. The intratumoral estrogen level may arise from local estrogen production in carcinoma cells or surrounding cells as well as from the uptake of peripherally generated estrogens, and so it has been debated whether the *in situ *estrogen production in the carcinoma cells is the major contributor to estrogen-stimulated tumor growth and thus may be a predictor of response to treatment with AIs [14,21-24]. Biochemically determined intratumoral aromatase enzyme activity has shown correlation with response to treatment with AI [25], and classic estrogen-dependent genes and proliferation markers changed in most tumors during treatment irrespective of response [26], supporting the hypothesis that *in situ *estrogen synthesis may be used as a predictive marker for response to treatment with aromatase inhibitor. Attempts have been made to measure the aromatase protein in the tumor tissue as an alternative to the biochemical assay. The aromatase protein has been detected in both fibroblasts, adipose cells, benign duct cells and invasive cancer cells and the results have been contradictory with respect to which cell types in the breast express the aromatase protein [27-33]. A promising new aromatase antibody (#677) has shown immunoreactivity in carcinoma cells, stromal cells or fibroblasts, adipocytes, normal epithelium, and inflammatory cells [31]. The multiplicative SIP score for carcinoma cells ("Proportion of aromatase immunopositivity" multiplied with "Relative intensity of aromatase immunoreactivity") was positively correlated with the aromatase enzyme activity measured by the biochemical tritiated-water release assay [32], indicating that aromatase expression in the carcinoma cells may be a predictive marker for response to AI treatment. Furthermore, since PR expression is induced by estrogen binding to ER, it may be hypothesised that high PR expression in combination with aromatase expression in primary tumor tissue identifies patients with superior benefit from AI treatment.

Due to the difficulties regarding determination of the aromatase protein expression and activity, cyclooxygenase-2 (COX-2) has been proposed as a surrogate marker for aromatase expression [34]. COX-2 is an isoform of the prostaglandin endoperoxidase synthetase that catalyzes key steps in the metabolism of arachidonic acid to prostaglandin E2 (PGE2), thereby regulating the synthesis of aromatase [35-37]: Expression of aromatase is controlled by unique promoters that lie upstream of tissue-specific exons. These first exons are alternatively spliced onto a common site upstream of the translation initiation codon in exon II. The aromatase mRNAs have unique 5'-untranslated regions, but the sequences encoding the aromatase enzyme are identical. In tumor tissue aromatase is up regulated by switching from the weak promoter I.4 to the strong promoters IIa and I.3. Switching from promoter I.4 to IIa and I.3 is likely to be mediated by the induction of COX-2 [38], which is expressed in a variety of cancers including invasive breast cancer [39-41].

The aim of this study was to explore whether the expression level of the aromatase protein in carcinoma cells alone or in combination with PR expression in the primary tumor may be predictive for response to treatment with the AI letrozole or the AE tamoxifen in the advanced breast cancer setting. The predictive utility of COX-2 for endocrine treatment as well as a surrogate marker for aromatase expression was also investigated. We have prepared tissue micoarrays (TMAs) and performed IHC analyses of ER, PR, COX-2 and aromatase in primary tumor tissue from 88 of the 907 patients who participated in the multicenter randomized phase III P025 trial which compared first-line tamoxifen or letrozole treatment in postmenopausal women with locally advanced or metastatic breast cancer [2,3]. A semi-quantitative IHC score has been determined for each of the four parameters. For aromatase expression, an evaluation of IHC staining in whole sections versus staining in TMAs was performed as previously described for ER, PR and COX-2 [42].

## Methods

This study was derived from the P025 study, an international, multicenter, double-blind, randomized, two-arm, phase III clinical trial comparing letrozole with tamoxifen as first-line treatment for postmenopausal women with advanced breast cancer [2]. The P025 study was conducted in accordance with the ethical principles that originated in the Helsinki II declaration and the appropriate local ethics review boards have approved the study, number by local ethical review board: 01025-KF12-138-99.

For this current sub-study it was possible to collect primary tumor material from 68 Danish patients and from 20 patients from the rest of Europe. Thus, altogether, paraffin blocks containing primary tumor material from 88 patients were collected. Sixty-five tumors were classified as invasive ductal and 12 as invasive lobular carcinoma. Three tumor samples were classified as neither lobular nor ductal and eight samples were not suitable for classification. Three of the 88 patients were originally allocated to a combination treatment of letrozole and tamoxifen. This combination therapy arm was stopped in the original P025 study and the patients were excluded from the intention to treat analysis, thus 85 patients are included in the present study, unless for comparison of aromatase expression in WS and TMAs where tissue from all 88 tumors were included. The end-point Time To Progression (TTP), is defined as described by Mouridsen *et al*. 2001 [2]. All patient data used in this study were provided by Novartis Phama AG, (Basel Switzerland) to the Danish Breast Cancer Cooperative Group (DBCG), following agreement with the departments who treated the patients.

### Immunohistochemical staining of ER, PR and COX-2

Sections, 3 μm, were dewaxed in coconut oil and rehydrated in a graded series of ethanol. Slides were preheated for 10 min. and boiled for 15 min. in microwave oven at 600 W in TEG buffer (pH = 9, Bie & Berntsen, Denmark) for antigen retrieval and rinsed in tap water. All immunostainings were performed at room temperature using the automated immunostainer Tech-mate 500 (Dako, Denmark), according to the following protocol: Slides were washed in TBS + 0.1% BRIJ-35 detergent (AX-LAB, Denmark) and incubated with primary antibody diluted in TBS + 0.1% BRIJ-35 + 1% BSA (Bovine Serum Albumin) + 15 nM sodiumazide for 60 min. After washing, endogenous peroxidase activity was blocked using 3% H_2_O_2 _in TBS + 0.1% BRIJ-35. The ChemMate EnVision^+ ^Detection Kit (Peroxidase/Dab, Rabbit/Mouse, K5007, Dako, Denmark) was used as detection system for the primary antibodies. After washing, slides were counterstained with haematoxylin and dehydrated in graded series of ethanol, and finally mounted with Pertex (Histolab, Denmark). The following primary antibodies, all monoclonal mouse subtype IgG_1_, were used: ER, clone ER1D5, 1:200 (Immunotech); PR, clone 16, 1:200 (Novocastra); COX-2, no 6111104, 1:150 (Cayman Chemicals). The specificity of the immunoreactions was verified by substitution of the primary antibody with the corresponding concentration of mouse IgG_1_, X 0931 (Dako, Denmark). In addition, positive control slides with breast tumor tissue or other tissue known to stain positive were included in every run. All stainings of the individual antigens were performed in a single run in order to minimize interserial staining variation.

### Immunohistochemical staining of Aromatase

All aromatase staining as well as evaluation of the staining was conducted by the author Hironobu Sasano. Sections, 3 μm, were stained and analyzed as described in Sasano et al. 2005 [32]. In brief: tissue sections were immunostained by a biotin-streptavidin method using a Histofine kit (Nichirei Co. Ltd., Tokyo, Japan). The antigen-antibody complex was subsequently visualized with 3.3'-diaminobenzidine solution (DAB) and counterstained with hematoxylin. Evaluation of aromatase IHC was performed by assessing the approximate percentage of carcinoma cell staining (proportion score) and classifying the proportion score values as the following: 0 = <1%, 1 = 1–25%, 2 = 26–50%, 3 = 51–75% and 4 = 76–100% immunopositive cells. The relative intensity of aromatase immune-positive cells was classified as follows: 0 = no immunoreactivity, 1 = weak, 2 = moderate and 3 = intense immunoreactivity. The SIP score is the proportion score multiplied with the relative intensity score. The evaluation of the aromatase immunoreactivity was performed without the knowledge of response to treatment.

### Preparation of tissue microarrays

TMA blocks were constructed using the TMA-builder from Histopathology Ltd. (AH-diagnostics, Denmark). Targets for arraying (areas with representative invasive tumor) were identified by marking the corresponding areas on haematoxylin-eosin stained sections from each paraffin block. Two tissue cores with a diameter of 2 mm were transferred from each donor block to the recipient TMA block. Kidney tissue was placed in the first core of the upper left and right corner of the TMA block to ensure correct orientation when examining the slides. All analyses were performed on TMAs [42], whereas analysis for aromatase was performed on whole sections (WS) as well.

### Evaluation of ER, PR and COX-2 immunohistochemical staining

All specimens were evaluated without knowledge of the clinical data. TMA slides were evaluated by light microscopy and the authors Katrine L. Henriksen scored all the samples and Birgitte B. Rasmussen has been consulted in cases of doubt. Only invasive tumor components were considered when assessing the staining. Semi-quantitative determination of ER and PR was performed according to the method described by Allred *et al*. [43]. This method was also used for semi-quantitative determination of the non-nuclear cytoplasmatic marker COX-2. In brief: The proportion of positive stained cells was judged as 0 = no cells stained, 1 = between 0 and 1% stained positive, 2 = between 1 and 10% stained positive, 3 = between 10 and 33% stained positive, 4 = between 33 and 66% stained positive, 5 = between 66 and 100% stained positive. In addition to the proportion score, an intensity score was made based on the average intensity of staining, 0 = negative, 1 = weak, 2 = intermediate and 3 = strong. The intensity score and the proportion score was added to obtain the total score referred to as the Allred score, which is 0 or between 2 and 8, 0 and 2 were interpreted as negative. Only nuclear staining was judged when scoring ER and PR, whereas cytoplasmatic staining was scored for COX-2.

### Evaluation of Aromatase immunohistochemical staining

Evaluation of aromatase staining in WS was done using the SIP score as described in Sasano et al. 2005 [32]. As described above, when preparing TMAs only areas containing primary tumor was used, thus instead of determining a SIP score the determination of "relative intensity of aromatase immunoreactivity" in carcinoma cells was scored for TMAs and not the "proportion of aromatase immunopositivity". Consequently no SIP score was obtained for the TMA sections.

### Statistical Analysis

Chi-square test was used to evaluate if the patients in the present study represent the entire study population of patients. The Kappa score was used to evaluate the agreement of aromatase immunointensity of carcinoma cells on whole sections (WS) versus TMAs, and for this analysis results from all 88 collected tumor samples were included. The Systematic difference between the scores on TMA and whole sections was analysed with Wilcoxon's Signed Rank Sum test on the difference (TMA-WS) and the Concordance is determined as the number of samples with no difference in scoring value in relation to the number of samples with a different scoring value and *P*-values for evaluation of WS versus TMA are given (Table 1). The association of aromatase expression with COX-2, as well as the agreement between expression of aromatase with ER and PR was done by calculating the Kappa score and *P*-values are given. Time to progression (TTP) was the primary endpoint in the P025 trial and was defined as the interval between the date of randomisation and the earliest date of disease progression. Kaplan-Meier plots were used to illustrate univariate survival and differences in TTP between subgroups were evaluated by the Wilcoxons rank sum test. A multivariate Cox proportional hazards model was used to investigate the effect of treatment and each of the parameters aromatase, COX-2 and PR, expressed as semi-quantitative IHC results. Interaction between treatment and aromatase expression was further investigated by subgroup analysis within each treatment group. When analysis of grouped scoring values was applied, scoring values were grouped as follows: For ER, PR and COX-2-expression "high" is defined as Allred score 7 or 8, "low" as Allred score 3 to 6 and "negative" as 0 or 2. For Aromatase, "high" is defined as SIP score 9 and 12, "low" as SIP score 1,2,3,4,6 or 8, and "negative" as SIP score 0 (See Table 2). In all tests, a *P-*value < 0.05 was used as level of significance.

**Table 1:** Comparison of carcinoma cell aromatase immunointensity in TMA and WS

**TMA versus WS n = 69**	**Concordance**	**Test for no difference**	**Kappa score**
	
	26%	(p < 0.002)	0.0048 (-0.080–0.17) (P = 0.41)

**Table 2:** Semi-quantitative IHC results: ER, PR, COX-2 and Aromatase

		Let n = 46	Tam n = 39	Total (n = 85)
**ER level (Allred score)**				

**negative**	≤ 2	3	2	5

**Low**	3	0	0	0
	
	4	1	1	2
	
	5	2	1	3
	
	6	6	3	9

**High**	7	10	8	18
	
	8	21	19	40

**N.A. (not assessable)**		3	5	8

**Total positive**		40	32	72

**PR level (Allred score)**				

**negative**	≤ 2	9	9	18

**Low**	3	3	1	4
	
	4	5	3	8
	
	5	4	5	9
	
	6	1	6	7

**High**	7	6	0	6
	
	8	14	12	26

**N.A. (not assessable)**		4	3	7

**Total positive**		33	27	60

**COX-2 level (Allred score)**				

**negative**	≤ 2	13	11	24

**Low**	3	0	2	2
	
	4	5	4	9
	
	5	10	2	12
	
	6	7	6	13

**High**	7	5	7	12
	
	8	1	0	1

**N.A. (not assessable)**		5	7	12

**Total positive**		28	21	49

**Aromatase level (Carcinoma cell SIP score)**				

**negative**	0	10	5	15

**Low**	1	3	3	6
	
	2	9	3	12
	
	3	2	3	5
	
	4	5	9	14
	
	6	8	5	13
	
	8	0	2	2

**High**	9	2	3	5
	
	12	2	1	3

**N.A. (not assessable)**		5	5	10

**Total positive**		30	30	60

## Results

### Patient data

The P025 trial recruited 907 patients and archived paraffin embedded tissue was collected from the primary tumor of 88 patients, of which 85 (9.4%) could be included in the clinical analyses [2]. As in the original study, the patients (85) are well balanced according to treatment allocation, 46 (54%) to letrozole and 39 (46%) to tamoxifen, age at randomization and Karnofsky Performance Score. However, the assessable patients differed from the total trial cohort with respect to having a significantly more advanced stage of disease at study entry and with respect to a larger proportion having received prior adjuvant treatment with tamoxifen (*P *< 0.01). In our subgroup of 85 patients the median TTP for letrozole treatment was 9.5 months (90% range: 0.33–39) and 9.5 months (90% range: 2.6–46) for tamoxifen. More detailed patient data are given in Henriksen et al. 2009 [44].

### Evaluation of aromatase staining in whole sections versus tissue microarrays

**Figure 1:**
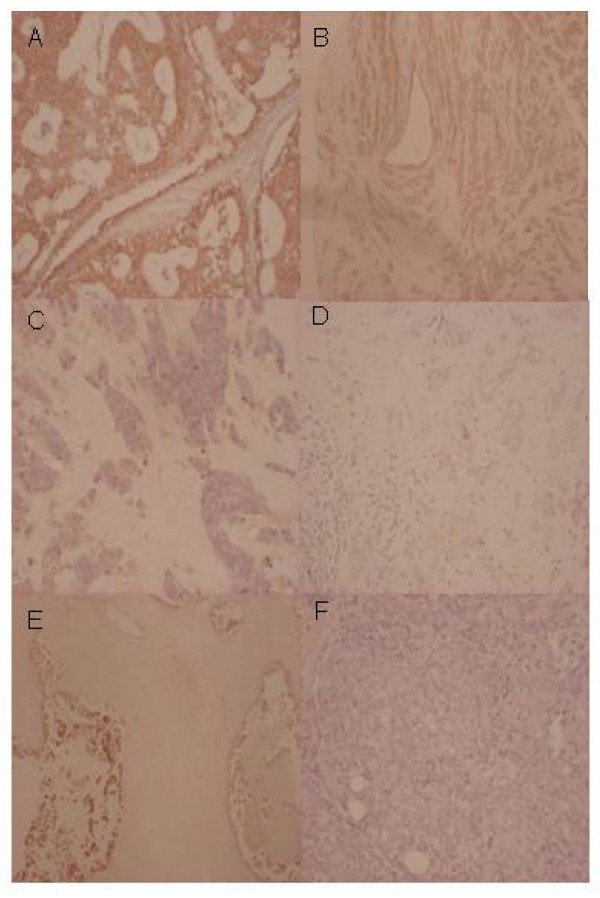
**Representative pictures of immunohistochemical aromatase staining of primary breast tumor tissue with the antibody #677**. **(A) **WS with high level of aromatase expression. **(B) **TMA with high level of aromatase expression. **(C) **WS with low level of aromatase expression. **(D) **TMA with low level of aromatase expression. **(E) **WS from a tumor showing heterogeneity in carcinoma cell staining intensity scored as high expression (score value 9). **(F) **A TMA illustrating discordant low intensity score (score value 1).

All of the collected 88 tumor samples were used for comparison, but for 19 of the samples either the WS or TMA results were missing due to technical reasons, thus 69 measurements were compared (Table 1). For 18 of the 69 tumors the same aromatase carcinoma cell intensity score was obtained from WS and TMA, resulting in a concordance of 26%. In 13 cases, the result obtained on TMA was higher than the result from WS, and for 38 of the samples WS scores were higher than TMA. A test for symmetry disclosed a systematic difference (*P *= 0.0013) and a significant difference was confirmed by the Signed Rank test (*P *< 0.002). We have further compared the expression level of carcinoma cell staining intensity by Kappa statistics and found that results obtained from WS and TMAs were not significantly associated (*P *= 0.41). Figure 1 shows examples of aromatase staining of breast tumor tissue from WS and TMAs. High aromatase expression in carcinoma cells is shown in Figure 1A and 1B, whereas 1C and 1D show low aromatase expression in carcinoma cells. Figure 1E and 1F illustrate a discordant staining result with high score in WS and low score in TMA.

### Scoring results of IHC staining of ER, PR, COX-2 and aromatase

ER, PR and COX-2 levels were scored by the Allred score on TMAs, as we have previously shown concordance between scores on TMAs and WS [42], whereas aromatase was analyzed on WS and evaluated by the SIP score. The semi-quantitative scoring results of ER, PR, COX-2 and aromatase are shown in Table 2. For all four markers the distribution of semi-quantitative scoring results were well balanced. The majority of tumors had a high Allred score for ER and PR. Only 6% of the tumors are scored negative for ER, 16% are scored low and 68% are scored high. The PR is more evenly distributed on the Allred scale, 21% of the tumors are scored negative, 33% low and 38% of the patients have a high expression of PR. None of the tumors were negative for both ER and PR. High cytoplasmic staining of COX-2 was observed in 15% of the samples, 42 % showed low COX-2 expression, and 28% were negative. The majority of tumours stained positive for aromatase, 61% with low expression and 9% were scored as high, whereas 18% was scored negative for the aromatase protein. For all markers technical problems impaired the scoring of between 7 and 12 of the tumor samples making them "not assessable" (N.A.).

### Association between aromatase expression and COX-2, ER and PR

To examine the association between tumor cell aromatase expression and the supposed surrogate marker COX-2, we performed Kappa statistics. Furthermore, the association between aromatase and ER as well as the estrogen-regulated protein PR was evaluated. Aromatase expression was not found to be associated with the expression of either COX-2 (*P *= 0.46) or PR (*P *= 0.83). However, a strong trend towards an association between tumor cell expression of aromatase and ER was found, (*P *= 0.057).

### COX-2 and aromatase expression for selection of treatment with either letrozole or tamoxifen

**Figure 2:**
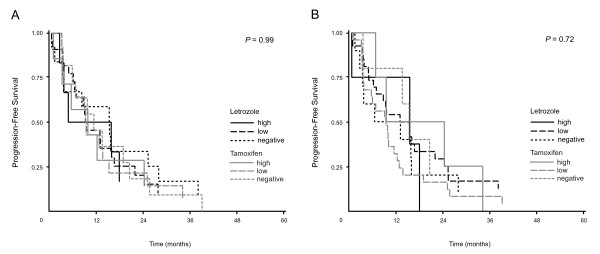
**Time to progression (TTP) according to high, low and negative expression of COX-2 and Aromatase in primary tumor tissue for patients treated with either letrozole (black) or tamoxifen (grey)**. **(A) *TTP according to COX-2 expression***: Letrozole treatment, COX-2 high, n = 6; COX-2 low, n = 22; COX-2 negative, n = 13. Tamoxifen treatment, COX-2 high, n = 7; COX-2 low, n = 14; COX-2 negative, n = 11. **(B) *TTP according to Aromatase expression***: Letrozole treatment, Aromatase high, n = 4; Aromatase low, n = 27; Aromatase negative, n = 10. Tamoxifen treatment, Aromatase high, n = 4; Aromatase low, n = 25; Aromatase negative, n = 4.

To evaluate whether our IHC analyses of either COX-2 or aromatase expression could be used to discriminate between TTP for patients treated with tamoxifen or letrozole, we compared TTP for all patients separated in subgroups of negative, low or high expression of the two markers. For each marker TTP was compared by uni-variate Wilcoxon-test and Kaplan-Meier plots as shown in Figure 2. Neither COX-2 nor aromatase expression was associated with superiority of either letrozole or tamoxifen in terms of TTP, *P *= 0.99 and *P *= 0.72 respectively. In agreement, a Cox analysis of COX-2 and aromatase expression level in relation to total endocrine treatment (letrozole- and tamoxifen-treated patients together) disclosed no difference in TTP between patients with negative, low or high marker expression, *P *= 0.95 and *P *= 0.94 for COX-2 and aromatase, respectively.

### Aromatase and PR expression for selection of treatment with either letrozole or tamoxifen

**Figure 3:**
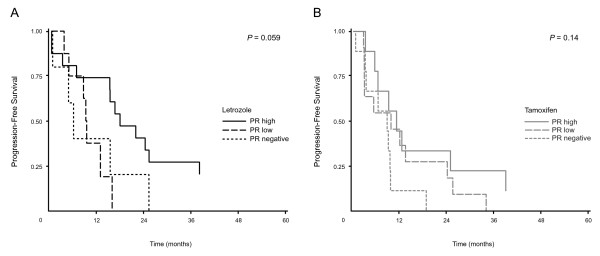
**Time to progression (TTP) according to high, low and negative PR expression in aromatase positive primary tumours from patients treated with letrozole or tamoxifen**. **(A) **Letrozole treatment, Aromatase positive, PR high, n = 16; Aromatase positive, PR low, n = 8; Aromatase positive, PR neg., n = 5. **(B) **Tamoxifen treatment, Aromatase positive, PR high, n = 9; Aromatase positive, PR low, n = 11; Aromatase positive, PR neg., n = 9.

As described above, we have compared the expression level of aromatase with ER and PR by Kappa statistics, and whereas the PR expression level was independent of aromatase expression, a strong trend towards association of aromatase and ER was found (*P *= 0.057). Thus, aromatase positivity combined with PR expression might add information regarding TTP for patients treated with tamoxifen or letrozole. When looking at all patients (letrozole- and tamoxifen-treated patients together), Cox analysis disclosed a significant difference between TTP for patients with aromatase positive tumors with negative, low and high PR content (*P *= 0.023), with high PR associated with long TTP. A further subgroup analysis of tamoxifen- and letrozole-treated patients showed a strong trend towards a different TTP for patients treated with letrozole (*P *= 0.059), but not for tamoxifen treated patients (*P *= 0.14), Figure 3A and 3B.

## Discussion

In this small retrospective sub-study of the P025 trial we have analyzed aromatase, COX-2, ER and PR expression by semi-quantitative IHC in primary tumor material from 9.4% of the patients included in the original study. The patients in our study were rather equally distributed regarding allocation to letrozole or tamoxifen. Our aim was to investigate whether aromatase- or COX-2 expression could be used as single markers for treatment, or if aromatase in combination with PR expression, could be used to select patients for treatment with either tamoxifen or letrozole. Furthermore, the utility of COX-2 as a surrogate IHC marker for aromatase as well as the feasibility of analysing aromatase expression by TMAs was evaluated.

In spite of the many resources and efforts applied to understand the basis of development, progression and prediction of response to treatment of breast cancer, only three predictive markers (ER, PR and HER-2) are recommended for routine IHC testing in breast cancer. Two of these, ER and PR, are predictive for response to endocrine treatment [45]. Since aromatase is the key enzyme in the estrogen synthesis and is expressed in both breast carcinoma cells, surrounding stromal cells as well as endothelial and benign breast cells [27,34], many attempts have been made to evaluate the importance of aromatase expression in the different cell types in the breast for local estrogen production and for utility as prognostic and predictive marker. The obtained results have been contradictory [26-29,33,46,47]. However, recent data have shown that the aromatase antibody #677 is very specific for IHC detection of the aromatase protein [31,32]. Although it has been assumed that the estrogen production in the surrounding stromal cells contributes significantly to the estrogen level within the carcinoma cells, it was found that only the IHC staining of aromatase in the carcinoma cells was correlated to the aromatase enzyme activity in the breast tissue [32]. Therefore, in this study we have firstly evaluated whether carcinoma cell aromatase detection with the #677 antibody gave comparable results in TMA and WS. The observed systematic difference and higher aromatase carcinoma cell staining intensity in WS compared to TMA can be explained by the marked heterogeneity of aromatase expression in the tumor tissue as illustrated in Figure 1E. Thus, the present study can not support TMA analysis of aromatase and accordingly, we have used the aromatase results obtained from WS when analyzing aromatase expression in relation to COX-2, ER and PR.

As mentioned previously, COX-2 is an enzyme that regulates the production of prostaglandins, which in turn regulate the synthesis of aromatase [36,37]. Several studies have found association between COX-2 expression and aromatase expression at the transcriptional level [35,36,48], and at the protein level using IHC [27,49,50], supporting that COX-2 may be used as a surrogate marker for aromatase content in tumor tissue [41]. This is in contrast to our finding, in which we compared the expression of COX-2 and aromatase in invasive carcinoma cells scored by semi-quantitative IHC and found no association between the aromatase expression (SIP score) and COX-2 expression (Allred score). We assume that the lack of association may be due to the complex regulation of the aromatase expression and activity, involving both COX-2 induced prostaglandins, but also many other stimulatory compounds e. g. cyclic-adenosine-monophosphate, glucocorticoids, interleukine-6 and tumor necrosis factor-α [26,51-57].

We found no association between carcinoma aromatase and PR expression, whereas a strong trend towards association between aromatase and ER in the carcinoma cells was found. This result is supported by Miki et al. who found no correlation between PR status and aromatase mRNA expression level in carcinoma cells and a significant association between aromatase mRNA and ER status in carcinoma cells [58]. This would imply that *in situ *estrogen production in the carcinoma cells is not the major source for ER-induced PR expression, an observation also supported by another recent study [46] in which aromatase, like ER, was expressed in inherently less aggressive tumors.

In carcinoma cells neither COX-2 nor aromatase expression alone were able to select groups of patients with superior benefit from tamoxifen therapy, but for patients with aromatase positive tumors a high PR expression identified a group of patients for whom endocrine treatment resulted in significantly longer TTP (*P *= 0.023). For letrozole treatment, patients with aromatase positive tumor cells and high PR expression had longer progression free survival (*P *= 0.059). This was not observed for patients treated with tamoxifen, indicating that in advanced disease the superiority of letrozole to tamoxifen is primarily observed in the group of patients with aromatase positive and high PR expression in the primary tumor cells.

## Conclusion

In summary, our results suggest that aromatase is too heterogeneously expressed in tumor tissue to allow detection in TMAs with 2 mm core size. COX-2 expression in breast carcinoma cells was not found to be a surrogate marker of aromatase expression, and neither COX-2 nor aromatase expression in carcinoma cells predicted response to letrozole or tamoxifen treatment. The lack of association between PR and *in situ *aromatase expression as well as no predictive value of aromatase expression for response to endocrine therapy, suggest that the estrogen synthesis in the carcinoma cells is not the major source for intratumoral estrogen. The strong trend towards an association of *in situ *carcinoma aromatase expression with ER expression may point towards aromatase as indicator of a good prognosis. In advanced disease, carcinoma cell aromatase expression in combination with high PR expression may select letrozole treated patients with longer time to progression.

## Competing interests

D. B. Evans is an employee and share holder of Novartis Pharma AG. The rest of the authors declare that they have no competing interests.

## Authors' contributions

AEL supervised the study and participated in drafting and writing of the manuscript. KLH carried out the IHC studies of COX-2, ER and PR and participated in drafting and writing of the manuscript. BBR supervised the IHC studies of COX-2, ER and PR and participated in writing the manuscript. HS carried out all IHC studies of aromatase. SM performed the statistical analysis. DE, BE and HM has participated in its design and coordination. All authors read and approved the final manuscript.

## Pre-publication history

The pre-publication history for this paper can be accessed here:

http://www.biomedcentral.com/1471-2407/9/185/prepub
